# Absorption and Transport Characteristics and Mechanisms of Carnosic Acid

**DOI:** 10.3390/biology10121278

**Published:** 2021-12-06

**Authors:** Xuexiang Chen, Meigui Huang, Dongmei Liu, Yongze Li, Qiu Luo, Katherine Pham, Minghong Wang, Jing Zhang, Runbin Zhang, Zhixi Peng, Xian Wu

**Affiliations:** 1 School of Public Health, Guangzhou Medical University, Guangzhou 510642, China; l1286955811@163.com (D.L.); liyongze15588@163.com (Y.L.); lq2833967441@163.com (Q.L.); wangminghong9913@163.com (M.W.); zingeui@gmail.com (J.Z.); z120635@163.com (R.Z.); pengzx0805@163.com (Z.P.); 2 Department of Food Science and Engineering, College of Light Industry and Food Engineering, Nanjing Forestry University, Nanjing 210037, China; huangmgnj@hotmail.com; 3 Department of Kinesiology, Nutrition, and Health, Miami University, Oxford, OH 45056, USA; phamk@miamioh.edu

**Keywords:** carnosic acid, Caco-2, absorption, transport, P-glycoprotein

## Abstract

**Simple Summary:**

Carnosic acid (CA), a phenolic diterpenoid mainly found in rosemary and sage, has been reported to possess various health-beneficial activities. However, detailed information about the absorption characteristics and mechanisms of CA and its tissue distribution still remains unclear. It has been well-recognized that the absorption, transport, and metabolism of dietary bioactive compounds are closely related to their biological functions. Herein, a mouse study and Caco-2 cell monolayer model of the intestinal epithelial barrier were used to understand the absorption and transport characteristics of CA. First, we determined the tissue distribution of CA in mice following oral gavage at a physiologically relevant dose. We found that CA was bioavailable systemically and present locally in the digestive tract, especially in the cecum and colon. Next, in Caco-2 cell monolayers, CA exhibited a moderate permeability and was subjected to mild efflux. Moreover, the apparent permeability coefficient of CA transported across Caco-2 cell monolayers was significantly changed when the inhibitors of specific active transporter and passive diffusion were added, suggesting that the absorption and transport of CA involved both passive and active transportation. The present study is an important first step towards understanding the absorption, transport, and metabolic mechanisms of CA.

**Abstract:**

Carnosic acid (CA) is a phenolic diterpenoid mainly found in rosemary and sage. CA has been reported to possess health-beneficial effects in various experimental settings. Herein, a mouse experiment and Caco-2 single-cell model were used to understand the absorption and transport characteristics of CA. First, we determined the tissue distribution of CA in mice, following an oral gavage at a physiologically relevant dose. We found that CA was bioavailable systemically and present locally in the digestive tract, especially in the cecum and colon. Next, we thought to characterize the absorption and transport of CA in the Caco-2 cell monolayer model of the intestinal epithelial barrier. In the Caco-2 cell model, CA exhibited a moderate permeability and was subjected to a mild efflux. Moreover, the apparent permeability coefficient (P_app_) of CA transported across Caco-2 cell monolayers was significantly changed when the inhibitors of specific active transporter and passive diffusion were added to cells, suggesting that the absorption and transport of CA involved both passive and active transportation. The present study is an important first step towards understanding the absorption, transport, and metabolic mechanisms of CA. This could provide the scientific basis for developing CA-containing functional foods or dietary supplements with improved bioavailability.

## 1. Introduction

**Figure 1:**
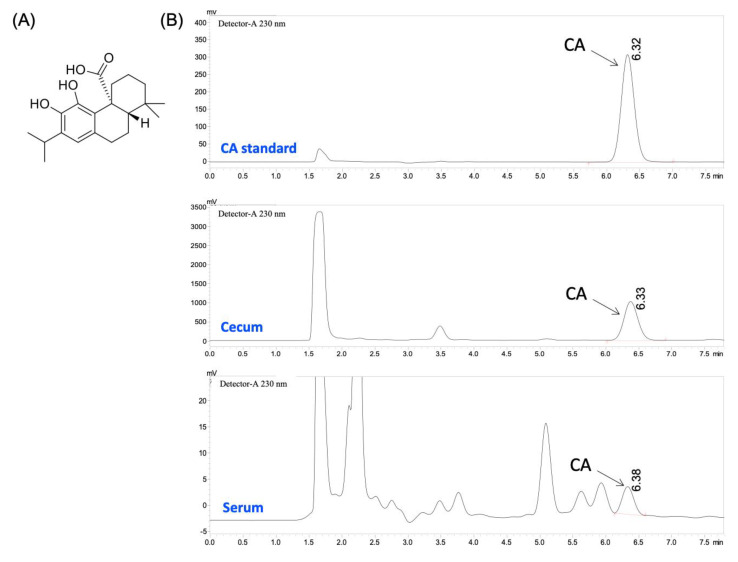
(**A**) Structure of CA. (**B**) Representative HPLC chromatograms of CA standard, serum and cecum samples.

Carnosic acid (CA, Figure 1A) is a bioactive phenolic diterpenoid mainly found in rosemary (*Rosmarinus officinalis L.*) and sage (*Salvia officinalis L.*) leaves [1]. CA has been reported to possess various health-beneficial activities including antibacterial [2,3], anti-tumor [4], anti-inflammatory [5], and neuroprotection [6]. For example, oral administration of 0.05% (*wt/wt*) CA for 5 weeks resulted in significant weight loss, improved glucose response, and reduced visceral adiposity and serum lipid levels in *ob/ob* mice [7]. Long-term oral administration of CA at 30 mg/kg/d improved diabetic nephropathy by upregulating the Nrf2/ARE pathway and suppressing the NF-κB pathway and was well-tolerated in mice [8]. Furthermore, administration of CA at 30 mg/kg/d in vivo and 4 μg/mL in vitro enhanced the suppressive effects of tamoxifen, an anti-cancer drug, and curcumin, the main active ingredient in turmeric, on breast cancer cells [9,10]. These observations suggest that CA can be used in functional foods or dietary supplements in humans for health promotion and disease prevention.

It has been well-recognized that the absorption, transport, and metabolism of dietary bioactive compounds are strongly related to their in vivo biological activities [11]. Yan et al. showed that CA was slowly absorbed in the small intestine of rats after oral gavage, and its circulating concentration was able to be maintained at 0.39 µg/mL 4 h after gavage [12]. Doolaege et al. found that oral administration of CA at 64.3 ± 5.8 mg/kg in rats resulted in predominantly free form in the bloodstream and its main elimination route was through fecal excretion [13]. In these two studies, an overall bioavailability of 40–65% was observed [11,13]. Given its bioavailability in animals, CA possesses great potential for preventing and/or treating human diseases. However, detailed information about the absorption characteristics and mechanisms of CA and its tissue distribution remains unclear. 

The Caco-2 cell monolayer model of the intestinal epithelial barrier is derived from human colon adenocarcinoma cells, which has the similar structure and function as human small intestinal epithelial cells, and its cell surface retains abundant drug-metabolizing enzymes and various transporters [14]. This model has been widely used to study the transport and absorption mechanism of orally ingested compounds [15]. Herein, we first determined the tissue distribution of CA in mice following oral gavage at a physiologically relevant dose. Then, we employed the Caco-2 single-cell model to investigate the absorption characteristics of CA. Specific inhibitors of passive and active transport, including verapamil, polyethylene glycols (PEG) 400, and EDTA, were applied to Caca-2 cell monolayers aiming to further illustrate the absorption and transport mechanism of CA. 

## 2. Materials and Methods

### 2.1. Animals, Cells, Reagents, and Instruments

Male ICR mice that are aged 5–6 weeks were purchased from Jiangsu JicuiYaokang Biotechnology (Taizhou, China) and kept in the Experimental Animal Center of Guangzhou Medical University (Guangzhou, China). The Caco-2 cell line and EMEM basic medium were purchased from ATCC (Rockefeller, MD, USA). The number of cell passages used in the experiment was between 20 and 40 generations. Penicillin-streptomycin, fetal bovine serum (FBS), pancreatin, and verapamil were purchased from Gibco (Grand Island, NY, USA). CA with ≥97% purity was obtained from Shanghai Aladdin Bio-Chem Technology (Shanghai, China). The 16-LC type HPLC and Wonda Sil C18-WR HPLC column were obtained from Shimadzu Instrument (Kyoto, Japan). The Transwell plate (12-well) was from Corning (Corning, NY, USA). The cell resistance meter was from Millipore Sigma (Boston, MA, USA). All other chemicals and reagents were of HPLC grade. 

### 2.2. Animal Experiment 

**Figure 2:**
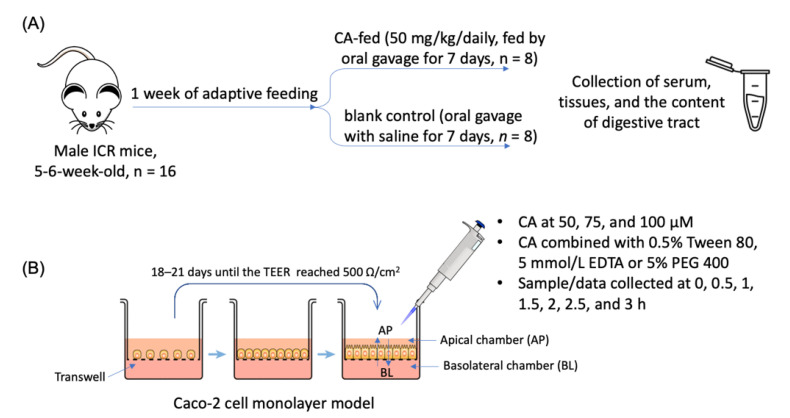
Study design of (**A**) animal experiment and (**B**) Caco-2 cell monolayer model.

The animal experiment protocol was approved by the Institutional Animal Care and Use Committee of Guangzhou Medical University (#GY2020-067, 30 July 2020). Male ICR mice aged 5–6 weeks were kept in a specific-pathogen-free environment with 12-h light/dark cycles. As shown in Figure 2A, after 1 week of adaptive feeding, the mice were randomly divided into a CA-fed (50 mg/kg/daily, fed by oral gavage for 7 days, *n* = 8) and a blank control (oral gavage with saline for 7 days, *n* = 8) group. Both groups were allowed to eat and drink ad libitum. Four hours after the last gavage, the mice were anesthetized, and their eyeballs were removed for blood collection. They were sacrificed and immediately dissected in the supine position. Serum, major organs (heart, liver, lung, stomach, spleen, kidney, small intestine, cecum, and colon), and the contents of the stomach, small intestine, cecum, and colon were collected, weighed, and stored at −80 °C. 

### 2.3. Determination of Tissue Distribution of CA in Mice by HPLC

Tissue samples and the contents of the digestive tract were homogenized in ultrapure water at 55 Hz for 8 min. Tissue/content homogenate (1 mL) or serum (100 μL) was mixed with 1 mL of 50% methanol, and the mixture was vortexed for 2 min. The mixture was extracted with ethyl acetate three times (6, 3, and 3 mL of ethyl acetate) and vortexed again for 2 min. The sample was centrifuged at 5000 rpm/min at 4 °C for 15 min, and the upper organic phase was dried under vacuum. Then, it was dissolved in 5% phosphoric acid methanol solution and filtered through a 0.22 μm microporous membrane. Concentrations of CA in serum, tissue, and content samples were determined using HPLC. CA standard was added to all samples, and the recovery rate was calculated. The chromatographic conditions for HPLC analysis were as follows: C18 chromatographic column: 5 μm, 150 × 4.6 mm; column temperature: 25 °C; mobile phase: methanol/0.1% phosphoric acid solution at 85%/15%; flow rate: 1.0 mL/min; detection wavelength: 230 nm; injection volume: 10 μL.

### 2.4. Establishment of Caco-2 Cell Monolayer Model

Caco-2 were cultured in EMEM complete medium containing 10% FBS, 1% non-essential amino acids, and 1% penicillin-streptomycin double antibody, and the culture conditions were 37 °C, 5% CO_2_, in a constant temperature incubator [16]. We first established non-toxic dose ranges of CA in Caco-2 cells using MTT assay. Dimethylsulfoxide (DMSO) was used as the vehicle to deliver CA to the cells. The final concentration of DMSO in all experiments was 0.1% (*v/v*) in cell culture media. Caco-2 cells were seeded in a 96-well plate at a cell density of 5 × 10^4^ cells/mL per well, and cultured under 5% CO_2_ and 37 °C for 24 h. A quantity of 200 μL of medium was added to each well to obtain a series of CA solutions (concentrations of 25, 50, 75, and 100 μmoL). After 48 h of treatment, Caco-2 cells were subjected to the MTT assay as described previously [17]. For the transport study, Caco-2 cells were seeded on the apical compartment (AP) of 12-well Transwell plates at a density of 2 × 10^5^ cells/mL. The complete culture medium was added into basolateral (BL) and AP compartments. The basolateral medium was replaced every other day.

### 2.5. Trans-Enterocyte Transport of CA in Caco-2 Cell Monolayer

As shown in Figure 2B, Caco-2 cells were incubated for 18–21 days until the transepithelial electrical resistance (TEER), measured using a cell resistance meter, reached 500 Ω/cm^2^. CA was dissolved in HBSS solution at 50, 75, and 100 μM. The HBSS solution containing CA combined with 0.5% Tween 80, 5 mmol/L EDTA or 5% PEG 400 was prepared. The cumulative migration (Q_r_), apparent permeability coefficient (P_app_), and the P_app_ value of bi-direction transport of CA were calculated according to formulas.

#### 2.5.1. Effect of Incubation Time and Dose on the Trans-Enterocyte Transport of CA

The 0.5 mL complete medium solution containing different concentrations of CA was added to the AP side of the 12-well Transwell plate, and 1.5 mL HBSS solution was added to the BL side. At 0, 0.5, 1, 1.5, 2, 2.5, and 3 h, 0.2 mL medium was taken from the BL side and stored at −80 °C. The TEER value of each Transwell chamber was measured.

#### 2.5.2. Effect of Verapamil, PEG 400, and EDTA on the Trans-Enterocyte Transport of CA

The 0.5 mL HBSS solution containing CA (50, 75, and 100 μM) combined with 100 μmoL/L verapamil, 5% PEG 400, or 5 mmol/L EDTA was added to the AP side of the Transwell plate, and 1.5 mL HBSS solution was added to the BL side. The effect of verapamil, Tween 80, PEG 400, and EDTA on the trans-enterocyte of CA was investigated according to the method described above. The TEER value of each Transwell chamber was measured.

#### 2.5.3. Determination of CA Concentration

The cryopreserved Transwell samples were transferred to a 15 mL centrifuge tube, and any remaining liquid was fully washed using 1 mL of 50% methanol solution which was also transferred to the 15 mL centrifuge tube. The mixture was vortexed for 1 min, and 6 mL of ethyl acetate solution was added to the tube and vortexed for another 2 min. The mixture was centrifuged at 6000 rpm/min at 4 °C for 10 min. The supernatant was dried under vacuum, dissolved in 5% phosphoric acid methanol solution, and filtered through a 0.22 μm microporous membrane. The concentration of CA was determined by HPLC as described above.

### 2.6. Statistical Analysis

All values were expressed as means ± standard deviations (SD). The data was statistically analyzed by SPSS 22.0 software (Chicago, IL, USA). Statistical significance between groups was determined by Student’s two-tailed *t* test (comparison between two groups) or an analysis of variance (ANOVA) (comparison between more than two groups) with significance levels of *p* < 0.05 or 0.01.

## 3. Results and Discussion

### 3.1. Tissue Distribution of CA in Mice after Long-Term Oral Administration

To mimic regular dietary consumption of CA in humans, mice were given 50 mg/kg/daily of CA by oral gavage for 7 days, which is generally achievable in humans via dietary supplements of CA. Based on equivalent surface area dosage conversion factors [18], the level we used in mice is equivalent to a daily dose of ~245 mg of CA for human intake in a 60 kg adult. This approach is different from the typical pharmacokinetic studies which involved animals that were gavaged or injected intravenously with a single or series of large dose of the test compound. Instead, CA was orally administered to mice at a physiologically relevant dose, which may better reflect the long-term regular dietary intake of CA in humans, thereby assuring the translational potential of the findings. Mice in both the control and CA-fed groups had free access to food and water throughout the entire experimental period. There was no significant difference between CA-fed and control mice in regard to body weight and the weights of the liver and spleen (Table 1). Further, we did not notice any apparent behavioral or appearance difference between the two groups. These observations suggest dietary intake of CA at the concentration of 50 mg/kg/daily was not associated with noticeable adverse effects. The results were consistent with previous reports that orally administered CA was nontoxic at even higher doses (64.3 and 90 mg/kg) [12,13].

HPLC was employed to determine the distribution of CA in serum, tissues, and the content of digestive tract in mice (Figure 1B). The recovery rates of these samples were between 79.1% and 113.0%. As shown in Table 2, we found that 15.22 μM of CA was detected in serum, which was similar to that of achieved in rats after a single-dose oral administration of CA at ~65–90 mg/kg/daily (=~39–54 mg/kg/daily in mice [12,13]). Previous reports showed that oral administration of other diterpenoids, such as carnosol [19] and 14-deoxy-11,12-didehydroandrographolide [20], resulted in a comparable circulating level of the ingested diterpenoid in vivo. Considerable levels of CA were also achieved in the heart, lung, and spleen (between 0.75 and 13.42 nmol/g). Interestingly, CA levels in the cecum and colon reached 2965.1 and 179.4 nmol/g tissue, respectively, comparing to a much lower level in the small intestine (1.87 nmol/g). The concentration of CA in cecal content was the lowest (0.15 nmol/g), as compared to gastric content (1.08 nmol/g), small intestine content (13.93 nmol/g) and colonic content (74.54 nmol/g). The hydrophobic nature of CA results in a poor bioaccessibility and bioavailability in vivo [21]. Many dietary bioactives are poorly absorbed in the small intestine. Thus, substantial quantities of unabsorbed compounds reach the cecum and colon, then extensively transformed by the gut microbiota [22,23]. These findings suggested that cecal microbiota in the large intestine might be mainly responsible for digestion and absorption of CA. Although the impact of CA on the composition of the intestinal microbiome has been reported in a few articles [24,25], the role of gut microbiota in the digestion and metabolism of CA remains unknown. This warrants future research. 

Previous studies showed that nearly no sulfate or glycoside conjugates of CA was present in the blood [13]. The focus was on the concentration of CA in these samples. Nevertheless, other Phase I and II metabolites of CA, e.g., glucuronide, oxidized and methylated derivatives [26], may exist and therefore requires further identification and quantification. Taken together, the snapshot of the tissue distribution of CA taken at 4 h after oral administration demonstrated that CA was bioavailable systemically and present locally in the digestive tract, especially in the cecum and colon. This finding suggests that the gut microbiome may play an essential role in the digestion and absorption of CA.

### 3.2. Trans-Enterocyte Transport of CA in Caco-2 Monolayer Model

**Figure 3:**
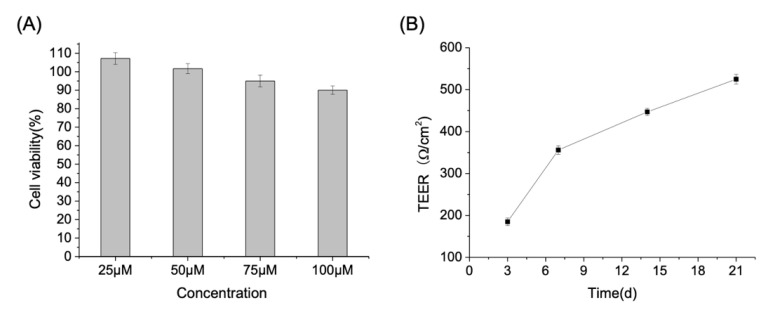
(**A**) Cell viability of Caco-2 after treatment with CA. (**B**) Changes of TEER value with time (day) in Caco-2 cell monolayer model. Data were expressed as mean ± SD.

Next, we employed the Caco-2 monolayer model of human intestinal epithelium to further understand the digestion and absorption processes of CA in the intestine [14]. First, we determined the non-toxic dose range of CA in Caco-2 cells using MTT colorimetric assay. The MTT result showed that CA did not affect the cell viability of Caco-2 cells at 100 μM (>90.01% viable), as compared to the untreated cells (Figure 3B). Non-cytotoxic concentrations were determined when the cell survival rate was above 90%. Thus, 50–100 μM of CA was chosen for the following in vitro experiments. The TEER value of the Transwell plate is a common indicator of the integrity of the Caco-2 single cell model [27]. Caco-2 cells were incubated for 18–21 days until the TEER reached 500 Ω/cm^2^ (Figure 3B). This indicated that a complete cell monolayer had been formed and could be used for transport experiments [28]. The apparent permeability coefficient (P_app_) is often used to measure the bioavailability of drug absorption in the Caco-2 cell model [29]. In this experiment, the P_app_ values of CA at 50–100 μM in both the AP-BL and BL-AP directions ranged 0.12–2.53 × 10^−6^ cm/s, indicating that CA is a moderately permeable drug [30] (Table 3).

**Figure 4:**
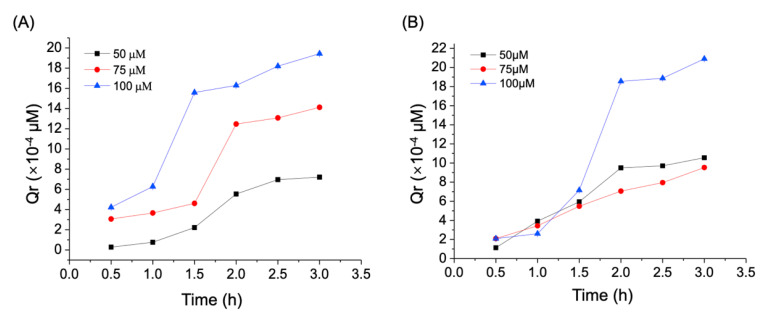
(**A**) Transmembrane migration of CA from AP to BL side. (**B**) Transmembrane migration of CA from BL to AP side. Data were expressed as mean ± SD.

Efflux ratio (i.e., P_app BL-AP_/P_app AP-BL_) is an indicator as to whether a compound undergoes active efflux across the cell monolayer. An efflux ratio > 2 indicates a significant involvement of apical efflux transporters. An efflux ratio falling between 1 and 2 typically suggests whether or not the test compound is the substrate of apical efflux transporters is uncertain [31]. Nevertheless, the transport mechanism can be determined by the use of specific inhibitors for membrane drug efflux transporters such as P-glycoprotein (P-gp), multidrug resistance associated protein 2 (MRP2), or breast cancer resistance protein (BCRP) [32]. As shown in Table 3, the efflux ratios of CA at 50 and 75 μM were all smaller than 1, except at 50 μM CA after a 3-hour incubation. When the concentration of CA reached 100 μM, the efflux ratios increased to 1.52–1.70 after 2-hour incubation. This suggests that lower doses of CA are mainly passively absorbed, but when the dose is increased to 100 μM it could undergo mild active efflux. A good correlation of drug absorption has been found between human small intestine and Caco-2 cells. These findings also confirmed that Caco-2 monolayer model is appropriate for investigating the absorption and transport mechanisms of CA [31]. Figure 4 shows the cumulative amount permeated (Qr) in the AP-BL (Figure 4A) and BL-AP (Figure 4B) directions. Qr values gradually increased as incubation time increased. The relation between the concentrations of CA and the Qr values was generally a linear relationship proportional to concentration, suggesting no saturation of the acceptor medium was observed in any concentrations and time points during the test time [33].

### 3.3. Effect of Verapamil and PEG 400 on the Trans-Enterocyte of CA

P-gp is a member of the ATP-binding cassette (ABC) transporter superfamily and is abundantly expressed in many human tissues and cells, including enterocytes and hepatocytes. P-gp is mainly present in the apical membrane of epithelia. This is where it mediates the efflux of a wide spectrum of substrates [34]. Many known drugs are substrates of P-gp, such as the antineoplastic drug vincristine, docetaxel, the antiarrhythmic drug digoxin, and the antibiotic clarithromycin. However, P-gp may pose a significant challenge in the application of drugs and dietary bioactive components as it decreases the absorption of that compound, thereby diminishing the therapeutic efficacy. Next, we sought to examine if P-gp plays a role in the absorption of CA in enterocytes. Verapamil and PEG 400 both have inhibitory effects on the function of P-gp efflux drugs. In the study of drug delivery, PEGs are often used as an oral absorption enhancer for drugs with low bioavailability [35,36,37,38]. As shown in Table 4, the addition of verapamil and PEG 400 to the cell culture media significantly enhanced P_app_ (AP to BL) of CA by 1.26- to 4.98-fold, as compared to the control group without verapamil or PEG 400. These results suggested that CA was indeed subject to active efflux and could be a substrate of P-gp. Besides P-gp, MRP and BCRP transporters are also members of the ABC superfamily [39]. Future studies are required to examine whether these transporters are involved in the absorption and transport of CA. In addition, the augmentation of P_app_ in the presence of verapamil or PEG 400 was inversely correlated with the concentration of CA, suggesting that mechanisms other than active efflux might be responsible for the transport of higher dose of CA.

### 3.4. Effect of EDTA on the Trans-Enterocyte of CA

The observations above revealed that there could be a considerable passive component in the transport of CA. EDTA is a metal ion chelator and can damage tight junction of the epithelia and increase the para-cellular permeability of hydrophilic molecules [40]. It was employed to determine whether CA has a paracellular route in the Caco-2 cell model. If passive diffusion is involved in the transport mechanism of CA, its transport would increase, which would follow the opening and/or destruction of the epithelial tight junctions. Interestingly, P_app_ (AP to BL) of CA was significantly increased by ~1.5-fold in the presence of EDTA when compared to the control group without EDTA (Table 4). These results indicated that the absorption and transport mechanism of CA in the Caco-2 cell monolayer involved passive diffusion. The amount of P-gp proteins is constant, and there will be transport saturation when the efflux rate of P-gp reaches a certain level. The transport rates of CA in Caco-2 cell monolayer generally increased with the concentration. This suggests that its transport mechanism might be primarily through the passive paracellular transport pathway. It is recognized that smaller, more hydrophobic compounds are more permeable and absorbed via passive transcellular or paracellular diffusion, while larger, more polar compounds are unlikely to exhibit good permeability via these routes. Given CA’s hydrophobic nature [41] and our in vitro data, we conclude that the absorption of CA largely occurs by passive diffusion. Nevertheless, active transportation of CA, especially at lower doses, might also considerably affect its absorption rate in the intestine. 

## 4. Conclusions

In this study, we determined the tissue distribution of CA in mice following oral gavage at a physiologically relevant dose and found that CA was primarily absorbed in the cecum and colon. In the Caco-2 cell model, CA exhibited a moderate permeability and was subjected to mild efflux. The results demonstrated that the absorption and transport of CA across Caco-2 cell monolayers mainly involved passive diffusion. The active transporter P-gp might also play an important role in the absorption of CA. Detailed information about the absorption characteristics and mechanisms of CA and its tissue distribution was never reported in the literature, which limits the application of CA in the food and nutraceutical industries. Thus, our study provides an excellent first step towards understanding the absorption, transport, and metabolic mechanisms of CA.

This study has limitations. Only one dose of CA was used in the animal study. Even though it is physiologically relevant, it may not necessarily fall into the optimal dose range of CA for health promotion. Moreover, Phase I and II metabolites of CA may exist at considerable levels, which were not determined in the present study. Moreover, the role of intestinal microbiota in the digestion and absorption of CA was not examined either. Lastly, the efflux ratio of CA combined with different inhibitors would provide further information about the transport mechanisms of CA in the intestine. Overall, future in vivo and in vitro studies using a wider dose range of CA are warranted to determine the detailed the absorption, transport, and metabolic mechanisms and the role of intestinal microbiota in the digestion and absorption of CA.

## Figures and Tables

**Table 1:** Body of mice. Data were expressed as mean ± SD.

	Body Weight (week, g)				
	1	2	3	4	5
Control	33.96 ± 1.2	35.1 ± 2.1	36.9 ± 2.3	38.5 ± 1.1	40.5 ± 2.2
CA	33.2 ± 0.9	34.8 ± 2.5	37.1 ± 1.8	38.9 ± 2.4	41.2 ± 3.1

**Table 2:** Concentrations of CA in serum, tissues, and content of digestive tract in mice. Data were expressed as mean ± SD.

Sample	Concentration (nmol/g or mL)
Serum	15.22 ± 0.02
Liver	10.56 ± 0.01
Kidney	0.66 ± 0.02
Stomach	35.98 ± 0.09
Small intestine	1.87 ± 0.00
Cecum	2965.10 ± 3.31
Colon	179.43 ± 0.26
Heart	0.75 ± 0.00
Lung	1.68 ± 0.00
Spleen	13.42 ± 0.01
Gastric content	1.08 ± 0.00
Small intestine content	13.93 ± 0.01
Cecal content	0.15 ± 0.00
Colonic content	74.54 ± 0.21

**Table 3:** Apparent permeability coefficient (P_app_) of CA in Caco-2 cell monolayer model. Data were expressed as mean ± SD.

Concentration of CA (μM)	Time (h)	P_app_ (10^−6^ cm/s)		Efflux Ratio
		AP-BL	BL-AP	
50	0.5	0.28 ± 0.07	0.12 ± 0.10	0.42
	1.0	0.36 ± 0.01	0.21 ± 0.09	0.58
	1.5	0.69 ± 0.09	0.36 ± 0.06	0.52
	2.0	1.11 ± 0.04	0.49 ± 0.07	0.44
	2.5	0.57 ± 0.03	0.56 ± 0.05	0.98
	3.0	0.22 ± 0.02	0.29 ± 0.03	1.32
	Average	0.53 ± 0.02	0.33 ± 0.03	0.62
75	0.5	1.03 ± 0.01	0.91 ± 0.02	0.88
	1.0	1.08 ± 0.04	0.86 ± 0.05	0.79
	1.5	0.83 ± 0.01	0.79 ± 0.07	0.95
	2.0	1.84 ± 0.10	0.62 ± 0.01	0.33
	2.5	0.83 ± 0.01	0.41 ± 0.03	0.49
	3.0	0.62 ± 0.01	0.38 ± 0.03	0.61
	Average	1.04 ± 0.05	0.66 ± 0.06	0.63
100	0.5	1.10 ± 0.10	0.39 ± 0.03	0.35
	1.0	1.32 ± 0.09	0.56 ± 0.04	0.42
	1.5	2.36 ± 0.08	2.53 ± 0.03	1.07
	2.0	0.87 ± 0.02	1.33 ± 0.05	1.52
	2.5	0.85 ± 0.05	1.45 ± 0.01	1.70
	3.0	0.80 ± 0.09	1.23 ± 0.03	1.53
	Average	1.20 ± 0.07	1.24 ± 0.04	1.03

**Table 4:** Apparent permeability coefficient (P_app_) of CA from AP to BL side in Caco-2 cell monolayer model with 100 μmoL/L verapamil, 5% PEG 400, and 5 mmoL/L EDTA. Data were expressed as mean ± SD. * denotes significant differences (*p* < 0.05, *n* = 3) compared to the CA group.

Concentration of CA (μM)	Time (h)	Group			
		CA	CA + Verapamil	CA + PEG400	CA + EDTA
50	0.5	0.28 ± 0.07	1.98 ± 0.06 *	3.76 ± 0.17 *	0.50 ± 0.01 *
	1.0	0.36 ± 0.01	1.89 ± 0.09 *	3.96 ± 0.10 *	0.24 ± 0.08
	1.5	0.69 ± 0.09	1.84 ± 0.06 *	2.35 ± 0.01 *	0.62 ± 0.02
	2.0	1.11 ± 0.04	2.08 ± 0.10 *	1.86 ± 0.35 *	0.92 ± 0.00 *
	2.5	0.57 ± 0.03	0.95 ± 0.03 *	1.89 ± 0.04 *	0.95 ± 0.02 *
	3.0	0.22 ± 0.02	1.37 ± 0.09 *	2.34 ± 0.01 *	1.49 ± 0.02 *
	Average	0.54 ± 0.05	1.50 ± 0.02 *	2.69 ± 0.04 *	0.79 ± 0.03 *
75	0.5	1.03 ± 0.01	1.12 ± 0.01 *	3.90 ± 0.10 *	0.92 ± 0.08
	1.0	1.08 ± 0.04	1.32 ± 0.01 *	2.99 ± 0.09 *	1.90 ± 0.08 *
	1.5	0.83 ± 0.01	0.85 ± 0.03	2.72 ± 0.46 *	1.94 ± 0.07 *
	2.0	1.84 ± 0.10	1.94 ± 0.06 *	2.35 ± 0.10 *	2.08 ± 0.03 *
	2.5	0.83 ± 0.01	0.87 ± 0.10	1.52 ± 0.06 *	1.28 ± 0.10 *
	3.0	0.62 ± 0.01	0.69 ± 0.01	1.19 ± 0.06 *	0.57 ± 0.01
	Average	1.04 ± 0.02	1.53 ± 0.04 *	1.45 ± 0.08 *	1.45 ± 0.05 *
100	0.5	1.10 ± 0.10	1.26 ± 0.02	3.21 ± 0.47 *	0.38 ± 0.00
	1.0	1.32 ± 0.09	1.70 ± 0.01 *	3.59 ± 0.10 *	2.25 ± 0.09 *
	1.5	2.36 ± 0.08	2.99 ± 0.03 *	2.89 ± 0.05 *	1.96 ± 0.07
	2.0	0.87 ± 0.02	1.24 ± 0.02 *	2.25 ± 0.02 *	3.01 ± 0.03 *
	2.5	0.85 ± 0.05	0.95 ± 0.05 *	2.44 ± 0.01 *	1.82 ± 0.08 *
	3.0	0.80 ± 0.09	1.09 ± 0.02 *	2.14 ± 0.02 *	1.56 ± 0.07 *
	Average	1.21 ± 0.03	1.53 ± 0.07 *	2.76 ± 0.07 *	1.83 ± 0.05 *

## Data Availability

Not applicable.

## Abbreviations

CA	carnosic acid
AP	apical side
BL	basolateral side
PEG	polyethylene glycol
P_app_	apparent permeability coefficient
Qr	cumulative migration
P-gp	P-glycoprotein
